# Virtual Specialist Care During the COVID-19 Pandemic: Multimethod Patient Experience Study

**DOI:** 10.2196/37196

**Published:** 2022-06-28

**Authors:** Katie N Dainty, M Bianca Seaton, Antonio Estacio, Lisa K Hicks, Trevor Jamieson, Sarah Ward, Catherine H Yu, Jeffrey D Mosko, Charles D Kassardjian

**Affiliations:** 1 North York General Hospital Toronto, ON Canada; 2 St Michael's Hospital Unity Health Toronto Toronto, ON Canada

**Keywords:** virtual care, specialist care, patient experience, COVID-19, medical care, virtual health, care data, decision support, telehealth, video consultation

## Abstract

**Background:**

Transitioning nonemergency, ambulatory medical care to virtual visits in light of the COVID-19 global pandemic has been a massive shift in philosophy and practice that naturally came with a steep learning curve for patients, physicians, and clinic administrators.

**Objective:**

We undertook a multimethod study to understand the key factors associated with successful and less successful experiences of virtual specialist care, particularly as they relate to the patient experience of care.

**Methods:**

This study was designed as a multimethod patient experience study using survey methods, descriptive qualitative interview methodology, and administrative virtual care data collected by the hospital decision support team. Six specialty departments participated in the study (endoscopy, orthopedics, neurology, hematology, rheumatology, and gastroenterology). All patients who could speak and read English and attended a virtual specialist appointment in a participating clinic at St. Michael’s Hospital (Toronto, Ontario, Canada) between October 1, 2020, and January 30, 2021, were eligible to participate.

**Results:**

During the study period, 51,702 virtual specialist visits were conducted in the departments that participated in the study. Of those, 96% were conducted by telephone and 4% by video. In both the survey and interview data, there was an overall consensus that virtual care is a satisfying alternative to in-person care, with benefits such as reduced travel, cost, time, and SARS-CoV-2 exposure, and increased convenience. Our analysis further revealed that the specific reason for the visit and the nature and status of the medical condition are important considerations in terms of guidance on where virtual care is most effective. Technology issues were not reported as a major challenge in our data, given that the majority of “virtual” visits reported by our participants were conducted by telephone, which is an important distinction. Despite the positive value of virtual care discussed by the majority of interview participants, 50% of the survey respondents still indicated they would prefer to see their physician in person.

**Conclusions:**

Patient experience data collected in this study indicate a high level of satisfaction with virtual specialty care, but also signal that there are nuances to be considered to ensure it is an appropriate and sustainable part of the standard of care.

## Introduction

At its broadest definition, virtual care is “the use of any technology (e.g., telephone, private messaging, videoconferencing) that supports health providers to collaborate with one another and to deliver remote care to patients” [1]. In response to the COVID-19 pandemic, the Ontario Ministry of Health released guidelines advising against direct patient care in nonurgent situations, and directed clinicians to transition the delivery of care to telephone- or video-enabled virtual visits online in early 2020 [2]. They also released unique billing codes to allow appropriate remuneration for these types of virtual visits [3]. Many professional colleges similarly encouraged their members to use virtual care wherever possible to minimize the risk of infection among their patients, especially those at higher risk of harm from COVID-19 infection [4,5]. In response to these guidelines and to ensure continuity of care, many hospitals and clinics shifted a majority of ambulatory visits to a virtual model. Increasing the ability to provide health care virtually not only supports social distancing and minimizes further potential spread of the virus but is also essential to ensure that patients continue to have access to medical guidance for nonemergent, but potentially serious, medical conditions. This is particularly true for urgent situations when the costs outweigh the benefits of bringing the person in for a physical visit (eg, immunosuppressed patients, those at high risk of infection, those with transportation issues).

Transitioning a large proportion of nonemergency, ambulatory medical care to virtual visits has been a massive shift in philosophy and practice that naturally came with a steep learning curve for patients, physicians, and clinic administrators. It has been widely reported that virtual care adoption has accelerated during the COVID-19 pandemic; however, published data remain limited. Previous studies have largely been limited to primary care, rural medicine, and nongeneralizable patient subgroups [6-8]. To provide optimal, safe care during the COVID-19 pandemic and beyond, we need to understand the key factors associated with successful and less successful experiences of virtual care. This is particularly important for areas newer to virtual care, such as specialist care delivery.

Increasingly, patient experience is recognized as an independent dimension of health care quality, along with clinical effectiveness and patient safety [9,10]. Accordingly, the aim of our study was to understand how patient experience data inform the development of guidance regarding characteristics associated with high satisfaction of virtual specialist care during the COVID-19 pandemic. This article specifically reflects our findings regarding the patient and family experience.

## Methods

### Study Setting

This study was conducted at St. Michael’s Hospital, a quaternary-care teaching and research hospital in downtown Toronto (Ontario, Canada), and part of the Unity Health system. As downtown Toronto’s adult trauma center, the hospital is a hub for neurosurgery, complex cardiac and cardiovascular care, complex medical specialty care, and care of the homeless and disadvantaged. Fully affiliated with the University of Toronto, St. Michael’s Hospital provides medical education to health care professionals in 29 academic disciplines.

### Study Design

This study was designed as a multimethod patient experience study using survey methods, descriptive qualitative interview methodology, and administrative data collected by the hospital decision support team.

### Ethics Approval

Institutional review board approval for this study was obtained from the Human Research Ethics Board of St. Michael’s Hospital (REB #20-198). All participants were given time to review the project information letter and provided written or verbal consent prior to the start of data collection. This report was compiled in compliance with the COREQ (Consolidated Criteria for Reporting Qualitative Research) guidelines for qualitative research reporting [11].

### Administrative Virtual Care Data

Deidentified administrative data on ambulatory visits (in-person, phone, and video) were retrospectively accessed via the admission-discharge-transfer systems at the hospital and categorized by clinical discipline (eg, neurology, orthopedic surgery) according to our internal coding structure. Visits to the emergency department, for day surgeries, and for diagnostic investigations other than those performed directly by a physician (eg, endoscopy) were excluded from the analysis.

### Sampling and Recruitment for Surveys and Interviews

A wide variety of ambulatory patients from both medical and surgical specialties were included in this study. Participating specialty departments included endocrinology, orthopedic surgery, neurology, hematology, rheumatology, and gastroenterology. This ensured that we were able to capture patients seen for a range of presenting complaints as well as appointment types. All patients who could speak and read English (or understand with help) and attended a virtual appointment in a participating clinic between October 1, 2020, and January 30, 2021, were eligible to participate.

Participants were recruited during their clinical visit with a participating physician. Following the appointment, the physician asked the patient if they would be willing to be contacted regarding a research survey and interviews about their experience with the virtual visit.

### Survey Data Collection and Analysis

During phase I, patient satisfaction with virtual visits was assessed through completion of an online survey made available through the SurveyMonkey platform (see Multimedia Appendix 1). At study initiation, there were no validated survey instruments to evaluate virtual medical care; thus, we adapted an instrument previously used to evaluate patient care visits provided through the Ontario Telemedicine Network [12]. The survey was only available in English due to time and resource constraints. The final question of the survey invited participants to provide their contact information if they would be willing to participate in a more in-depth telephone interview.

Survey data results are reported as simple counts or percentages. Logistic regression of variables was not possible owing to the low sample size.

### Interview Data Collection and Analysis

Patients and families who consented to participate in an interview via the online patient survey (described above) were contacted by the research coordinator. Interviews were conducted by telephone to support social distancing restrictions and avoid geographic bias in recruitment.

The interviews followed a semistructured format using an interview guide informed by the study objective and addressing key topics, including understanding of the patient’s functional status, health concerns, and experience of the virtual visit with their specialist (see Multimedia Appendix 2). The selection of follow-up questions, question order, and phrasing varied according to each participant’s narrative. This approach enabled the emergence of participant-led accounts, reflecting their varied histories, modes of expression, and foci of experience. All interviews were conducted by an experienced qualitative interviewer (MBS), audio-taped, and transcribed verbatim by an external transcription service. The qualitative data were managed using NVivo (NVivo 12, QSR International Pty Ltd) qualitative software. The interview transcripts were supplemented with field notes to collect data that were not captured on audiotape (eg, dynamics, emotional aspects, contextual factors).

In keeping with the iterative process of qualitative methodology, data analysis occurred in conjunction with data collection to continuously monitor emerging themes and general areas for further exploration. We used a thematic analysis approach, as described by Braun and Clarke [13], to enable the identification of patterns and meaning across the sample. The analysis was led by two members of the research team with extensive qualitative expertise (KND and MBS) with regular collaboration with the rest of the study team. We extracted and collated the interview sections that reflected the key areas of interest and carried out the initial coding process. We then used the emergent codes to guide a de novo analysis of the entire corpus for overarching subthemes and used NVivo to record which subthemes occurred in each interview, ensuring their accurate representation in the analysis. Subthemes that expressed similar experiential patterns were brought together to develop the themes that we felt best represented the participants’ perspectives. Versions of the analysis were reviewed with the research team at regular intervals, and the final analytic framework was discussed among all authors until we reached consensus on its validity and applicability. We employed the following techniques to support the analytic rigor and trustworthiness of our analysis: comparison of coding between analysts, seeking alternative explanations for the data during development of the final analytic framework, and interrogating the coherence of interpretations through discussion with the research team [14].

## Results

### Virtual Care at St. Michael’s Hospital During the Study Period

The quantitative analysis results of virtual visit volumes at the study center are first described to provide context for the survey and interview findings. During the study period from April to December 2020, there were 593,172 total ambulatory visits at St. Michael’s Hospital, 50.76% (n=301,105) of which were conducted virtually. Of note, only about 5176 visits (approximately 1.72%) were conducted virtually in the period of 2018-2019, before the pandemic, at the study hospital. In the specialty departments that participated in this study, 93,920 total ambulatory visits were conducted, over 50% of which were performed virtually. Among the virtual visits, over 96% were by phone. Some disciplines, notably orthopedic surgery and rheumatology, did not adopt virtual care as robustly as other specialties such as neurology and endoscopy. Neurology also had a much higher adoption of video visits than the other specialties, which may be attributed to their need for visual examination. Ambulatory volumes across disciplines were not linked to the uptake of virtual visits overall or by video specifically. A summary of the visit types for each specialty that participated in this study is provided in Table 1.

**Table 1:** Visit data for participating specialties (April 1, 2020, to January 30, 2021).

Specialty	Total visits, n	In-person visits, n (%)	Virtual visits		
			Total, n (%)	Phone visits, n (% of total, % of virtual)	Video visits, n (% of total, % of virtual)
Endoscopy	25,115	6992 (27.84)	18,163 (72.32)	18,012 (71.72, 99.39)	111 (0.44, 0.62)
Orthopedics	17,033	13,568 (79.66)	3465 (20.34)	3462 (20.33, 99.91)	3 (0.02, 0.09)
Neurology	18,774	6309 (33.60)	12,435 (66.34)	10,802 (57.54, 86.66)	1663 (8.87, 13.37)
Hematology	10,303	4413 (42.83)	5890 (57.17)	5889 (57.16, 99.83)	1 (0.01, 0.02)
Rheumatology	7224	4419 (61.17)	2805 (38.83)	2702 (37.40, 96.33)	103 (1.43, 3.67)
Gastroenterology	15,471	6517 (42.12)	8954 (57.88)	8815 (56.98, 98.45)	139 (0.90, 1.55)
Total	93,920	42,218 (44.95)	51,702 (55.05)	49,682 (52.90, 96.09)	2020 (2.15, 3.91)

### Survey and Interview Sample

Between October 2020 and January 2021, 216 patients from the seven participating clinics at St. Michael’s Hospital completed the virtual care experience survey. The large majority of the sample had attended their virtual visit for a follow-up appointment (vs an initial visit or emergency situation) and had previously attended a virtual medical appointment. Almost half of the sample was over 65 years of age and almost 60% identified as female. In the same time period, 125 patients agreed to participate in an interview and 18 patients with diverse characteristics, health conditions, and virtual visit types were selected for interviews. Detailed demographics of the participants are provided in Table 2.

**Table 2:** Survey and interview participant demographics.

Demographic characteristics		Survey (n=216), n (%)	Interviews (n=18), n (%)
**Gender**			
	Female	125 (58.1)	11 (61)
	Nonbinary	1 (0.5)	0 (0)
**Education**			
	High school	27 (12.7)	4 (22)
	Postsecondary diploma/degree	81 (43.6)	9 (50)
	Graduate degree	39 (18.3)	3 (17)
	Health care professional	8 (3.7)	0 (0)
	Professional school	23 (10.6)	2 (11)
**Age (years)**			
	18-34	14 (6.5)	1 (6)
	35-54	53 (24.8)	7 (39)
	55-80	125 (60.8)	10 (56)
	80+	17 (7.9)	0 (0)
**Location of birth**			
	In Canada	131 (61.2)	12 (67)
	Outside Canada	83 (38.8)	6 (33)
First virtual care visit		68 (31.9)	11 (61)
**Type of appointment**			
	First visit with specialist	19 (8.9)	4 (22)
	Follow-up visit	181 (85.4)	14 (78)
	Other	12 (5.7)	
	Phone call	186 (86.9)	13 (72)
	Video call	25 (11.6)	3 (17)
	Telephone and video call	0 (0)	2 (11)

### Survey Results

Survey respondents overwhelmingly had a very positive experience with virtual care. Almost 87% of people surveyed indicated that their virtual visit had been conducted by telephone (rather than video conference). They reported feeling comfortable connecting with their physician virtually, felt the physician spent sufficient time with them, and that their privacy was respected during the virtual call. Very few (3.8%) needed help with their virtual appointment or experienced technical difficulties during the visit (6.6%). However, despite 93% of respondents being satisfied with their virtual care experience, 50% still reported that they would prefer to see their physician in person if it were safe to do so. In addition, only 68% felt that the physician-patient relationship was the same as during an in-person visit.

When asked more generally about their opinion of virtual care, 25% were still unsure if virtual care is an acceptable way to provide health care for an initial consultation, but the majority agreed it was acceptable for follow-up visits (86%) and to discuss test results (85%). Full survey results are provided in Table 3 and Table 4.

**Table 3:** Survey results for questions scored on a 5-point scale (N=216).

Survey questions	Strongly disagree (1), n (%)	Disagree (2), n (%)	Neither agree nor disagree (3), n (%)	Agree (4), n (%)	Strongly agree (5), n (%)
I was comfortable connecting with my physician virtually (phone/video)	2 (0.9)	0 (0)	5 (2.3)	78 (36.6)	128 (60.1)
My privacy was respected	2 (0.9)	0 (0)	5 (2.4)	58 (28.0)	142 (68.6)
I felt that my physician spent sufficient time with me	3 (1.4)	3 (1.4)	6 (2.8)	65 (30.4)	137 (64.0)
My telephone/video assessment was thorough	2 (0.9)	3 (1.4)	17 (8.1)	81 (38.4)	108 (51.2)
I left the virtual visit with a clear understanding of the next steps	2 (0.9)	4 (1.9)	8 (3.8)	74 (34.9)	124 (58.5)
Compared to an in-person visit, the physician-patient relationship was the same	4 (1.9)	29 (13.7)	35 (16.5)	70 (33.0)	74 (34.9)
Having a virtual visit saved me time	2 (0.9)	3 (1.4)	13 (6.1)	55 (25.9)	139 (65.6)
I experienced technical difficulties during my appointment	132 (62.3)	59 (27.8)	7 (3.3)	10 (4.7)	4 (1.9)
I needed help with my virtual visit from a family member or friend	154 (72.6)	40 (18.9)	10 (4.7)	4 (1.9)	4 (1.9)
If it were safe to do so, I would prefer to meet with my care provider in person	14 (6.6)	19 (9.0)	72 (34.0)	62 (29.3)	45 (21.2)
I was satisfied with my virtual visit	2 (0.9)	2 (0.9)	10 (4.7)	85 (40.3)	112 (53.1)

**Table 4:** Survey results for questions scored on a 3-point scale.

Survey questions	Agree, n (%)	Disagree, n (%)	Not sure, n (%)
A virtual visit is an acceptable way to provide care for an initial consultation (N=211)	77 (36.5)	83 (39.3)	51 (24.2)
A virtual visit is an acceptable way to provide care for a routine follow-up appointment (N=214)	185 (86.5)	13 (6.1)	16 (7.5)
A virtual visit is an acceptable way to discuss test results (N=214)	182 (85.1)	5 (2.3)	27 (12.6)
A virtual visit is an acceptable way to provide an urgent follow-up assessment (N=216)	118 (55.4)	48 (22.5)	47 (22.1)

### Interview Results

#### Overview

We interviewed 18 patients who had a minimum of one virtual specialist appointment at St. Michael’s Hospital. The majority of interview participants were female, ranged in age from 45 to 64 years, and over one third reported having seen more than one type of specialist virtually during the COVID-19 pandemic (Table 2). Overall, participants were extremely happy with the opportunity to connect virtually with their physicians and were generally very satisfied with the appointments conducted. Our qualitative data analysis revealed three key themes that provide a deeper understanding of participants’ experience of virtual care: (1) the impact of improved access, (2) influence of the nature of the visit, and (3) consideration of the nature of the medical condition.

#### Impact of Improved Access

Due to the nature of St. Michael’s Hospital as a large quaternary-care academic health science center, people frequently travel from outside Toronto to see the specialty physicians affiliated with the hospital. Many of the patients we spoke with mentioned that their high satisfaction with virtual care was driven by being able to access their specialists without the nuisance of the potentially long trip to the city.

Yeah, it’s great. Because just for us to go to Toronto, you know, there’s always an overnight. Because I can’t go there and back in one day. It becomes an expensive journey just to go to the hospital for a follow-up.

Participant 6

The COVID-19 pandemic appeared to amplify preexisting travel challenges for patients who were very wary of travelling to a large urban center for fear of exposure to the virus. Participants told us that it was important to be able to access their specialists despite the pandemic and the option of virtual appointments met that need. They expressed significant concerns about the risk to themselves and their caregivers/family members of exposure to SARS-CoV-2 from coming into the city in person, and were very grateful for the opportunity to keep their appointments using the virtual medium.

I don’t see how the consult I had on the phone was going to be any different than going to the place. In fact, it felt safer right now during a pandemic to not have to leave the house and not have to go into the hospital.

Participant 17

#### Influence of Reason for the Visit

Many of the participants had seen more than one type of specialist during the pandemic, and thus were able to draw on significant experience of virtual care during the interviews. Participants told us they felt that virtual care is acceptable for certain types of appointments such as initial consults that are conversation-based (to discuss and explain requirements for further tests, examinations, and procedures), routine follow-up for stable conditions, to review tests results, and whenever the interaction between the patient and specialist would mostly be question and answer–based to support the provision of information and advice. However, for appointments that would typically involve a physical examination, when they are experiencing pain, or if their health condition has progressed or changed, they explained that they would prefer to be assessed and have an opportunity to speak with their specialist in person. For these types of appointments, participants perceived virtual care to come with higher potential for misinterpretation or a misdiagnosis than in-person care, and that this could ultimately impact the trajectory of their treatment.

So, I think it depends on the appointment. If it’s just a routine follow-up to go through test results, that’s fine. But if it’s an actual, “Hey, you know I’m not feeling well. This is what is going on,” I’d prefer in person so they can actually touch it or see it.

Participant 5

It’s good but I’m really not sure if what she’s seeing is right, because it’s different than what I’m feeling. And I think that part was a little bit frustrating or worrisome, because I don’t want something to be inaccurately marked and somehow – like that has the potential to affect my care.

Participant 10

Conversely, patients spoke about virtual appointments being more efficient for themselves and their physicians. Some mentioned the time savings of not having to take time off work or other responsibilities for routine follow-up appointments, and noted that their physicians seemed to be “more prepared” for the virtual appointments than they typically were for previous in-person visits. It was felt that this greater familiarity with the patient’s chart and recent test results made for a better discussion about their state of health and current care needs.

It’s not only that [referring to time savings], but I’m actually more confident. Because when XX phones me, she has reviewed the file, knows what she’s going to say, and off I go. Previously, I would sit there while she reviewed the file on the screen and got up to date on it. This way, she gets up to date on her own speed, and when I talk to her, it’s usually a very brief interview. She tells me what she sees. I ask her questions and it’s over. No, no. This is much, much better.

Participant 1

#### Considering the Nature and Status of the Medical Condition

Several interview participants had long, complicated medical histories and chronic condition(s). For the most part, these participants still felt that the virtual appointments had met their care needs, and told us they were satisfied with both the quality of care received and the interaction they had with their physicians. However, these were the same patients who most frequently expressed some hesitation around virtual care, explaining that they did not believe that the specific health issues they have, including rashes, vision-related problems, and tremors, could be assessed clearly through video or adequately described by phone.

Because rheumatology is such a hands-on profession, I think they can’t assess your joints at all over Zoom or OTN [Ontario Telemedicine Network]. I find that their rheumatologists are probably missing quite a bit because they can’t get the information that they would normally get. The neurologist…it’s probably also missing that physical component because they can’t assess your tremors, or your eye tracking, or your reflexes. They can’t do any of that. The conversations have been kind of restricted to things like which drugs we’re on…like which drugs I’m on and kind of skipping over that physical component.

Participant 13

For the participants managing comorbidities and complex chronic health problems, an annual check-up appointment by telephone or online was perceived to be acceptable so long as their conditions remain stable. To discuss changes in their health status and treatment options, these patients definitely preferred the option to see their physician in person.

I know my condition well. If everything is going well and I am stable then the phone appointments are fine. When there are specific flare-ups or…my blood work is off for too long – then he needs to see me.

Participant 15

## Discussion

### Principal Findings

Using survey and qualitative interview methods, we examined the experiences of patients accessing virtual specialist care at a quaternary-care center in Toronto, Ontario, Canada. Our results indicate that, overall, patients were very satisfied with the quality and efficiency of virtual care, and value it as an option for safe and equitable access to specialist care. However, our data also revealed nuances to that value, which are important to take into account as we consider virtual care as a permanent care delivery option.

There is an increasing number of publications on the virtual experiences of patients, most of which have been conducted in singular fields such as oncology, pre- and postnatal care, and psychiatric care [15-20]. Similar to our findings, in the existing literature, there appears to be a consensus that virtual care is a satisfying alternative to in-person care, with benefits such as reduced travel, cost, time, and infectious exposure, and increased convenience [15-20]. Our analysis further revealed that the specific reason for the visit, and the nature and status of the medical condition are important considerations in terms of guidance on where virtual care is most effective. Previous barriers or challenges identified also included technological issues, a potential lack of personal connection, inability to perform physical and visual assessments, and inequities in access [20,21]. Although some of these align with our data, we did not hear specifically about technological issues or a lack of personal connection. This likely can be attributed to the fact that the majority of the visits in our study sample were conducted by telephone (vs video or a new virtual platform), and that many of our participants had longstanding relationships with their specialists and therefore the personal connection may be stronger. The remuneration of both telephone and video care at equivalent rates in Ontario, Canada, may play a factor in the lack of uptake of video conferencing given reports from other jurisdictions (ie, telephone may be perceived as more cost-efficient) [22,23].

A few findings were notable in our data. First, as mentioned above, a large majority of “virtual” visits reported by participants were conducted by telephone, which is an important distinction from previous reports. Virtual care has been defined as:

any interaction between patients and/or members of their circle of care, occurring remotely, using any forms of communication or information technologies, with the aim of facilitating or maximizing the quality and effectiveness of patient care [24].

However, it seems that discussion of virtual care often assumes a technology- or video-based component. Other terms that have been used in this literature include “telehealth” or “telemedicine,” which may be more representative of the preferred medium. This finding is noteworthy in terms of unpacking held assumptions about what is possible with virtual care and understanding existing virtual care infrastructure. The availability, familiarity, and technical ease of telephone-based care (for both the provider and recipient) likely contributed to the overall predominance of telephone visits. Further investigation is required to determine the relative advantages and drawbacks of the different modalities for delivering virtual care.

Second, despite the positive value of virtual care discussed by the majority of interview participants, 50% of the survey respondents still indicated they would prefer to see their physician in person. This could reflect the fact that at the time of the survey, virtual care may have still been seen as a COVID-19–related intervention rather than a potentially permanent option for care delivery. In addition, only 68% of respondents felt that the physician-patient relationship was the same as during an in-person visit. These findings highlight that there still may need to be a cultural shift before there is complete comfort with virtual options as part of the standard of care in Ontario.

Our study adds to the growing literature on virtual care in the era of the COVID-19 pandemic and helps to inform virtual care implementation beyond the pandemic as well. As the pandemic has evolved, it has become clear that enhanced infection prevention protocols are likely to remain at some level in ambulatory care [25]. Moreover, now that the benefits of virtual care have been experienced by patients and physicians, it is likely that demand for virtual care will continue beyond the pandemic. As such, we need to use experience data such as those presented here to understand how virtual care performs in real time from both the perspective of those who access care and those who deliver it. Based on our data, we recommend a flexible, blended care model utilizing virtual care and in-person visits based on the type of appointment (eg, new patient, routine follow-up, assessment of new problem), patient preference, travel burden, and infection considerations. One size will not fit all, and a blended model combining in-person and virtual visits when tailored to each patient and visit is consistent with a patient-centered approach to care delivery. Virtual visits (including telephone visits) appear to be particularly well-suited for routine follow-up appointments focused on nonurgent matters. Virtual visits are also valued by patients when travel is either too costly or burdensome. However, further work is required to delineate the balance between telephone- versus video-based virtual care and to determine which types of care visits are most effective virtually. As new virtual technologies and systems emerge, such as secure texting and remote monitoring, it will also be important to reevaluate the benefits and drawbacks of each approach, and to ensure that privacy, confidentiality, and data quality are maintained. Prior to COVID-19, virtual care was largely managed as a distinct service model from in-person services; however, it is clear from learnings throughout the pandemic, including our work, that virtual and traditional care are complementary and that patients need the flexibility to seamlessly transition between both modalities. Considerable further work around quality, safety, convenience, preference, and appropriateness needs to occur so that the decisions on what modalities to offer and in what circumstances are evidence-informed. We must also evaluate the impact of virtual care on health care equity, as not all patients will have access to the same technology, and we must ensure that socioeconomic disparities are not widened or exacerbated by the adoption of virtual health care options.

### Strengths and Limitations

This study represents a robust and diverse sample of patient responses, including diversity in gender, immigrant status, and specialty care provided. We also leveraged the strength of multiple data collection methods to be able to both capture the experience of a large number of patients while at the same time being able to gather a deeper understanding through the individual interviews.

Despite its strengths, the study does have some limitations. We only performed the data collection within a single hospital site located in downtown Toronto, Canada. While our results may not be generalizable to more rural or remote locations, we do feel that our site represents a fairly typical tertiary health science center, and therefore the insights learned here should be useful to other centers.

Use of a web-based survey prevented us from recruiting patients without an email address, which may have biased our sample toward respondents with higher digital literacy and educational attainment. The survey and interviews were only conducted in English, which also may have introduced a language bias to the sample. Both of these methodologic choices may also explain a lower survey sample size than we expected. In addition, as with all patient-reported and qualitative data, there is some level of volunteer bias. That said, the survey was offered to all patients who participated in a virtual visit (physicians did not hand select who would receive the survey). Volunteer bias in surveys and interviewing is almost impossible to avoid; however, we used rigorous qualitative methodology to ensure we recruited a balanced and saturated sample for the interviews. Lastly, the predominance of telephone and follow-up visits in our data, while reflective of the “real-life” use of virtual care during the pandemic, limits the ability to draw conclusions about video visits or other care settings (eg, urgent care).

### Conclusion

Providing alternative ways for providers and patients to deliver and access high-quality specialist care has become a necessity during the COVID-19 pandemic; however, the need and preference for virtual care are likely to only increase in the future. The patient experience data captured in this study indicate a high level of satisfaction with virtual specialist care, but also signal that there are nuances to be considered to ensure it is an appropriate and sustainable part of the standard of care. This type of multimethod, patient-oriented research combined with provider experience insight will be crucial in informing realistic guidance for health care systems across Canada.

## Appendix

Multimedia Appendix 1 Study survey.

Multimedia Appendix 2 Patient interview guide.

## Abbreviations

COREQ: Consolidated Criteria for Reporting Qualitative Research
