# Supplementary material for: Virtual Specialist Care During the COVID-19 Pandemic: Multimethod Patient Experience Study
Source: JMIR Med Inform. 2022 Jun 28;10(6):e37196. doi: 10.2196/37196 (PMC9239568; doi:10.2196/37196)
Supplement: Multimedia Appendix 1 [file medinform_v10i6e37196_app1.pdf]

Patient Experience of Virtual Care (Unity Health)

Study Letter of Information & Consent

Welcome to the Virtual Care Survey!

You are being asked to consider participating in a research study because you have recently received your care “virtually” (either over the phone or by video).

Background/Purpose

During the COVID-19 pandemic, most physician offices were closed to in-person visits, and visits were done either over the phone or through video. This was a sudden and unexpected change for both doctors and patients, with little time to prepare.

We are interested in learning about the patient experience with virtual care (telephone or video) during the COVID-19 pandemic, particularly things that went well or did not go well with your virtual visit. We hope to use the results from the survey to generate recommendations for health-care providers. The research is being conducted by a group of specialist physicians at St. Michael’s Hospital.

Description of the Study

If you decide to participate, you will be asked to complete a 10-minute online survey that is completed anonymously. Survey questions focus on the type of interaction, the aspects that were positive and negative, and how virtual care compares to traditional in-person visits. Participation in this study is voluntary. You may decline to answer any questions that you do not wish to answer and you can withdraw your participation at any time by not submitting your responses.

Potential Harms and Potential Benefits

There are no known harms associated with participation in this study. There are also no direct benefits to you for participating in this study, other than the potential to help improve virtual care for yourself and other patients in the future.

Participation and Withdrawal:

Participation is voluntary. If a question is not applicable to you or you feel uncomfortable answering please complete them to the best of your ability. We would appreciate your responses to all questions, none of the individual questions are mandatory. Your consent to participate in this study is demonstrated by your voluntary completion and submission of the survey. Due to the anonymous nature of the survey (e.g. there is no way to link your name to the responses), you cannot retract your survey once you have submitted your responses.

Confidentiality and Privacy

It is important for you to know that any information that you provide will be confidential. All of the data will be summarized and no individual could be identified from these summarized results. Furthermore, the web site is programmed to collect only responses and will not collect any information that could potentially identify you (such as machine identifiers). Completed surveys will be kept confidential, and will be retained until study completion, after which the surveys will be securely destroyed. Access to the study data will be limited to the study investigators and their delegates, and the Unity Health Toronto Research Ethics Board for the purposes of monitoring the study. When information is transmitted over the internet confidentiality cannot be guaranteed. Survey Monkey stores electronic data on servers in the United States, whose privacy laws differ from those in Canada. The data entered may be subject to the PATRIOT Act that allows authorities to request access to these records without your notification. Survey data will be deleted off of the Survey Monkey servers within 6 months of the completion of the study. We will not use or save this information without your consent. If you prefer not to submit your survey responses through this host, please contact your physician, so you can participate using an alternative method.

The results of this study may be presented at conferences, seminars or other public forums, and published in journals. If you would like to receive a copy of the results of this study, please contact either investigator.

If you have any questions regarding your rights as a research participant, you may contact the Chair of the Unity Health Toronto, Research Ethics Board at 416-864-6060 ext. 2557 during business hours.

If you have any questions about the study, contact any of the investigators listed below.

Charles Kassardjian - Charles.Kassardjian@unityhealth.to
Jeff Mosko - Jeff.Mosko@unityhealth.to
Katie Dainty - katie.dainty@utoronto.ca

1. Please enter your response here:

- ◯ I consent to participate in this study
- ◯ I do not wish to participate (please close your web browser now).

2. On average how long would it take you to get to St. Michael's Hospital for your clinical appointment if you were to come in person (travel both ways)?

3. Which type of specialist/doctor did you see today?

4. What type of appointment is this?

- ◯ My first visit with this doctor
- ◯ A follow-up visit with this doctor
- ◯ Other (please specify)

5. How was your virtual visit conducted?

- ◯ By telephone
- ◯ By video
- ◯ By both telephone and video
- ◯ Other (please specify)

6. Was this the first time you have had a virtual visit?

- ◯ Yes
- ◯ No
- ◯ Unsure

7. Thinking about your last virtual visit with your doctor, please rate your level of agreement with the following statements:

	1 = Strongly Disagree	2 = Disagree	3 = Neither agree nor disagree	4 = Agree	5 = Strongly Agree
I was comfortable connecting with my physician virtually (phone and/or video)	○	○	○	○	○
My privacy was respected.	○	○	○	○	○
I felt that my physician spent sufficient time with me.	○	○	○	○	○
My telephone/video assessment was thorough	○	○	○	○	○
I left the virtual visit with a clear understanding of the next steps (eg a treatment plan)	○	○	○	○	○
Compared to an in-person visit, the physician-patient relationship was the same.	○	○	○	○	○
Having a virtual visit saved me time.	○	○	○	○	○
I experienced technical difficulties during my appointment (e.g. video connection problems, lack of voice clarity with phone reception)	○	○	○	○	○
I needed help with my virtual visit from a family member or friend.	○	○	○	○	○
If it were safe to do so, I would prefer to meet with my care provider in person	○	○	○	○	○
I was satisfied with my virtual visit	○	○	○	○	○

8. Please read the following statements and indicate whether you agree or disagree:

	Agree	Disagree	Not sure
A virtual visit is an acceptable way to provide care for an initial consultation	◯	◯	◯
A virtual visit is an acceptable way to provide care for a routine follow-up appointment	◯	◯	◯
A virtual visit is an acceptable way to discuss test results	◯	◯	◯
A virtual visit is an acceptable way to provide an urgent followup assessment	◯	◯	◯

Thank you for answering those questions. Now we would like to collect a bit of information about you to help us analyze the results of the survey. This survey is anonymous and no identifying information will be collected. If you are completing this for someone else, please enter the information that is accurate for the patient.

9. What is your age?

- ◯ Under 18
- ◯ 18-24
- ◯ 25-34
- ◯ 35-44
- ◯ 45-54
- ◯ 55-64
- ◯ 65-80
- ◯ 80+

10. What gender do you identify with?

- ◯ Male
- ◯ Female
- ◯ Non-binary
- ◯ Prefer not to answer

11. What is the highest level of education you have completed?

- ◯ No formal education
- ◯ Elementary school
- ◯ High school
- ◯ College diploma
- ◯ Undergraduate degree
- ◯ Graduate degree
- ◯ Health care professional designation
- ◯ Professional school
- ◯ Other (please specify)

[ ]

12. Where were you born?

- ◯ In Canada
- ◯ Outside Canada (please specify what Country)

[ ]

13. What is the language you prefer to communicate in?

- ◯ English
- ◯ French
- ◯ Other (please specify)

[ ]

14. As part of this study we are also interested in talking with patients and their families to understand your experience in more detail. **If you would be willing to hear more about participating in an interview (approximately 30 mins), please enter your name and contact information below.** A member of the research team will contact you with more information. If you do not wish to participate in an interview, please enter "No" in the name category.

Name [ ]

Email Address [ ]

Phone Number [ ]

That is the end of the survey! Thank you very much for your time and for sharing your opinion with our research team. We hope our research will help improve virtual care for all patients in the future.
