# Supplementary material for: Virtual Specialist Care During the COVID-19 Pandemic: Multimethod Patient Experience Study
Source: JMIR Med Inform. 2022 Jun 28;10(6):e37196. doi: 10.2196/37196 (PMC9239568; doi:10.2196/37196)
Supplement: Multimedia Appendix 2 [file medinform_v10i6e37196_app2.docx]

**APPENDIX B - Patient Interview Guide**

Introduction: Just before we get started, I would like to confirm that you have consented to this interview and are comfortable moving forward?

As a reminder, you can skip any questions you do not wish to answer, and the interview will be audio recorded to ensure we capture everything you want to tell us.

The purpose of this study is to learn about your experience with your most recent virtual care visit at Unity Health. I’m particularly interested in hearing about what you thought about how it went and if and how it could have been improved.

1. To start, tell me a little bit about yourself and why you were seeing the doctor? We really just would like to know if this is the first time you are seeing this doctor if this is a follow-up visit.
2. Did you speak to the doctor on the phone or by video?
3. What were your initial thoughts when you heard you would be talking to the doctor on the telephone/by video?
4. Tell me about how you thought the appointment went?
5. Was there anything you thought was better than your in-person appointments?
6. Was there anything that you thought could have been better about the appointment?
7. Is there anything else you would like to tell me today about your experience with virtual care?
